# Bilateral Comparisons of Quadriceps Thickness after Anterior Cruciate Ligament Reconstruction

**DOI:** 10.3390/medicina56070335

**Published:** 2020-07-03

**Authors:** Joo-Hyun Lee, Soul Cheon, Hyung-Pil Jun, Yu-Lun Huang, Eunwook Chang

**Affiliations:** 1 Department of Kinesiology, Inha University, Incheon 22212, Korea; joohyun09@gmail.com (J.-H.L.); soul9879@gmail.com (S.C.); 2 Institute of Sports and Arts Convergence (ISAC), Inha University, Incheon 22212, Korea; 3 Department of Physical Education, Dong-A University, Busan 49236, Korea; hjun@dau.ac.kr; 4 Department of Kinesiology, University of Wisconsin-Eau Claire, Eau Clair, WI 54702, USA; huangyul@uwec.edu

**Keywords:** anterior cruciate ligament reconstruction, quadriceps, vastus intermedius, muscle atrophy

## Abstract

*Background and objectives*: Anterior cruciate ligament reconstruction (ACLR) often results in quadricep atrophy. The purpose of this study was to compare the bilateral thickness of each quadricep component before and after ACLR. *Materials and Methods:* Cross-sectional study design. In 14 patients who underwent ACLR, bilateral quadricep muscle thicknesses were measured using a portable ultrasound device, 1 h before and 48–72 h after ACLR. Two-way analysis of variance (ANOVA) was used to compare muscle thickness pre- and post-ACLR between the limbs. *Results:* The primary finding was that the vastus intermedius (VI) muscle was significantly smaller in the reconstructed limb after ACLR compared to that in the healthy limb (Reconstructed limb; RCL = Pre-operated (PRE): 19.89 ± 6.91 mm, Post-operated(POST): 16.04 ± 6.13 mm, Healthy limb; HL = PRE: 22.88 ± 6.07, POST: 20.90 ± 5.78 mm, F = 9.325, *p* = 0.009, η^2^p = 0.418). *Conclusions:* The results represent a selective surgical influence on the quadricep muscle thickness. These findings highlight the need of advanced strengthening exercises in order to restore VI thickness after ACLR.

## 1. Introduction

The anterior cruciate ligament (ACL) injury is a common sport related injury that influences high performance athletes and recreational athletic populations with up to 250,000 ACL ruptures annually [1]. Anterior cruciate ligament reconstruction (ACLR) is considered as a primary treatment option for ACL injury. Although ACLR restores the structural deficiency, patients who have undergone ACLR frequently show quadricep atrophy and muscle loss [2,3]. A study revealed that quadricep atrophy substantially leads to chronic joint problems, such as joint arthritis [4]. Quadricep strength reduction and muscle mass loss also negatively influence not only sports participation, but also daily life activities. Several previous magnetic resonance image (MRI)-based investigations reported the consequences of decreased muscle volume and knee function reduction. In addition, measured quadricep muscle atrophy resulted in quadricep weakness during rehabilitation after ACLR, which led to negative consequences such as post-traumatic knee osteoarthritis for ACLR patients [5,6]. Therefore, it could be important to prevent quadricep atrophy due to ACLR.

The quadriceps perform various functions to allow human movement by providing knee joint stabilization and a cushioning effect [7]. In addition, the quadriceps play a significant role in explosive movements and injury prevention during sports [8]. In general, the quadriceps are considered as one large muscle group that is specifically divided into five muscle components, including the rectus femoris (RF), vastus intermedius (VI), vastus lateralis (VL), and vastus medialis (VM) and vastus medialis oblique (VMO). Although the primary function of the quadriceps as a whole is thought to be knee extension, each component of the quadriceps contributes differently for executing certain tasks [9]. For example, the VI and VM cross sectional areas (CSAs) were strongly correlated with the knee extension-maximum voluntary isometric contraction [10,11,12] A close relationship between VI thickness and knee extension force has been shown [13]. These results suggested further investigations regarding the influence of each quadricep component on human performance.

It has been desirable to compare the quadriceps on the reconstructed and non-reconstructed sides of patients who have undergone ACLR. Bilateral comparison is important for deciding the return to activity after ACLR. Many studies have reported that less than 15% bilateral differences in knee extension strength could be a proper standard for the return to activity [14,15,16]. Additionally, various studies have suggested the bilateral comparison of the functional performance limb symmetry index (LSI), such as the hop-test LSI, as a decision-making assessment for the return to activity [17]. Furthermore, the quadricep morphology (muscle volume and CSA) was reported to be closely related to functional performance, which leads to the speculation of the significance of muscle size [18,19,20]. Thus, bilateral muscle thickness comparison could be necessary to predict patients’ functional restoration after ACLR.

Muscle atrophy typically occurs from disuse for a long period of time. Many studies regarding quadricep morphological alterations and functions after ACLR have been conducted in patients in who were 8–12 months past post-reconstruction [3,21,22]. A recent study showed that quadricep thickness was decreased within a week after surgery [23], which represents the possibility of muscle morphology alteration in a relatively short period of time rather than from long-term disuse. Therefore, the present study aimed to compare the bilateral thickness of each quadricep component before and after ACLR. While many previous investigations reported the bilateral quadricep muscle morphology, indicating significant atrophy of the reconstructed limb, these studies investigated long-term post-surgical muscle thickness (7~29 months post-ACLR) [24,25,26]. In the short term, post-surgically, since there could be a direct physiological influence on the quadriceps on the reconstructed limb after ACLR [23], we hypothesized that the quadricep thickness in the reconstructed limb would be lesser than that in the healthy limb.

## 2. Materials and Methods

### 2.1. Patients

Fourteen patients who underwent unilateral ACLR (10 males and 4 females, age = 30.4 ± 5.9 years, mass = 69.9 ± 10.8 kg, height = 170.8 ± 8.6 cm, and body mass index = 23.8 ± 2.3 kg/m^2^) voluntarily participated in the study (Table 1). A priori power analysis was conducted utilizing G*Power software to determine a minimum number of participants. For effect size calculation, we thoroughly reviewed many previous studies that investigated quadriceps morphological alteration following ACLR [3,25,27]. Then, we calculated Cohen’s d effect size and found ranges of large effect (d = 1.21–2.11). In order to calculate the sample size of repeated analysis of variance for two groups, we utilized Cohen’s f with large effect size (f = 0.4), significance at an alpha level of 0.05, and actual power (1-β) of 0.8 and the minimum sample was detected as fourteen patients.

Before the ACLR, the patients were informed regarding the current investigation by the orthopedic surgeon. Once the patients agreed to the study participation, the researcher explained the details of the study on the day of ACLR. Patients who had the acute ACL injury and radiologically confirmed complete ACL rupture were included in the study. Patients were excluded in the case of a history of myopathy, previous knee surgery, previous lower back surgery, or rheumatological diseases. All patients signed informed consent after they understood the study process sufficiently. The study protocol was approved by the university Institutional Review Board (Study ID:180226).

### 2.2. Study Design

**Figure 1:**
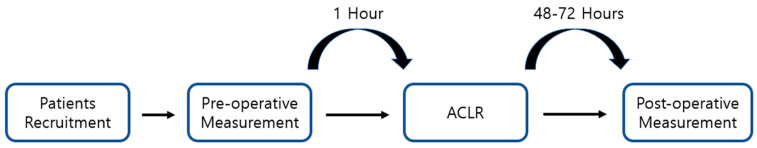
Overview of the study design demonstrating the preoperative and postoperative measurements. ACLR: Anterior cruciate ligament reconstruction.

The current study was developed with a cross-sectional observational design following the Strengthening the Reporting of Observational Studies in Epidemiology (STROBE) guidelines [27]. An overview of this study design is presented in Figure 1. Before surgery, the patients filled out a participation agreement. The researcher then conducted the ultrasound measurements (reconstructed limb: RCL, healthy limb: HL). Bilateral quadriceps were measured an hour before surgery and within 48–72 h after surgery. The surgical procedure was conducted by one orthopedic surgeon for all subjects. The average time for utilizing tourniquet was 67 min. In order to minimize edema by ACLR, all patients received general post-surgical care including cryotherapy, compression, and elevation.

### 2.3. Muscle Thickness Measurement

**Figure 2:**
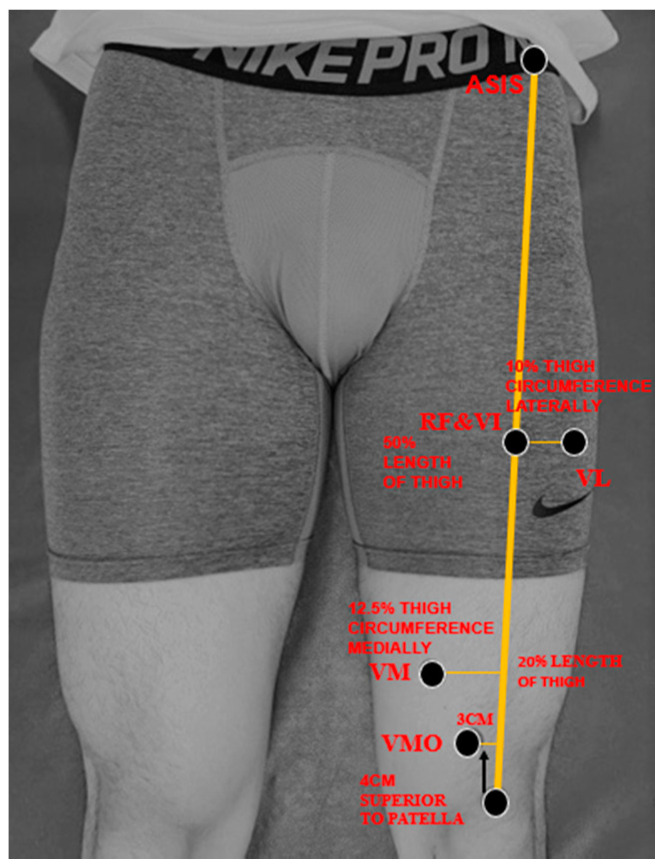
Quadricep thickness measurement sites. ASIS: anterior superior iliac spine; RF: rectus femoris; VI: vastus intermedius; VL: vastus lateralis; VM: vastus medialis; VMO: vastus medialis oblique.

A cross-sectional research design was used to compare quadricep thickness between the RCL and HL before and after ACLR. Quadricep thickness was measured an hour before and 48–72 h after the reconstruction by using a portable ultrasound device (7.5 MHZ transducer, Healcerion, Seoul, Korea). Previous investigations reported excellent intra-rater (ICCs 0.95–0.97) and acceptable-good inter-rater (ICCs 0.62–0.90) reliability of quadriceps thickness measurement using ultrasound [28,29]. To measure the thickness of each quadricep component, the patients were positioned supine and hip external rotation prevented. The thickness of each quadricep component was measured in a random order. The location of the measurement site of each component was based on a previous study [30] (Figure 2). Specifically, the measurements were made along with the length of the thigh from the superior pole of the patella to the anterior superior iliac spine (ASIS). The RF and VI were measured at 50% of the length between the superior pole of the patella and the ASIS [30]. The VL was measured laterally, at 10% of patient’s thigh circumference from the RF and VI measurement sites. The VM was measured medially, at 12.5% of the patient’s thigh circumference from 20% of the length the line between the superior pole of the patella and the ASIS. The VMO was measured 4 cm superior and 3 cm medial from the border of the patella [31]. The image was adjusted until the muscle boundary was visible on the screen and the depth of the image was measured when the femur was centered on the screen. The ultrasound images were recorded three times per muscle by a single examiner. Once all the muscle thicknesses were measured, the images were saved for further analysis.

### 2.4. Image Analysis

**Figure 3:**
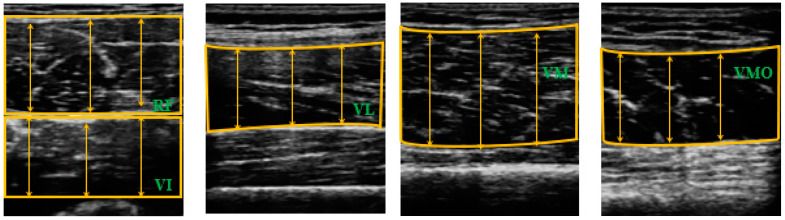
The images used to measure the muscle thicknesses of the components of the quadriceps.

ImageJ software (National Institutes of health, Bethesda, MD, USA) was used to analyze each muscle thickness. The images were recorded when the boundaries of the muscles were clear and the femur was visible from the center of the screen. The subcutaneous adipose tissue and bone tissue were identified, and the distance between them was defined as muscle thickness [32]. The RF thickness was defined as the distance between the superficial border of the muscle and the deep border of the muscle. The VI thickness was defined as the distance between the superficial border of the muscle and the superficial border of the femur. The VL, VM, and VMO thicknesses were defined as the distances between the superficial and inferior borders of the muscles [31] (Figure 3). Muscle thickness was defined as the average value of three lines placed equally spaced in the muscle belly; the three lines were measured three times each and the average value obtained (Figure 3). The average value of the three images was utilized in the statistical analyses.

### 2.5. Statistical Analysis

The Kolmogorve–Smirnov test was conducted to assess the data normality and it was confirmed that the data were normally distributed (*p* > 0.05). The effect of limb reconstruction on bilateral quadricep thickness differences was evaluated using a 2 (time: pre- and post- reconstruction) × 2 (limb: RCL and HL) repeated measure of analysis of variance (Two-way ANOVA). When there was a significant interaction effect, we utilized paired T-tests between pre- and post-results and independent-T test between limbs. Effect sizes were reported by squaring partial eta (η^2^p) and the η^2^p was classified as 0.01 = small, 0.06 = medium, and 0.14 = large [33]. Statistical analysis was conducted using the SPSS 25.0 software for Windows (IBM INC, Chicago, IL, USA) with statistical significance set a priori at α ≤ 0.05.

## 3. Results

**Figure 4:**
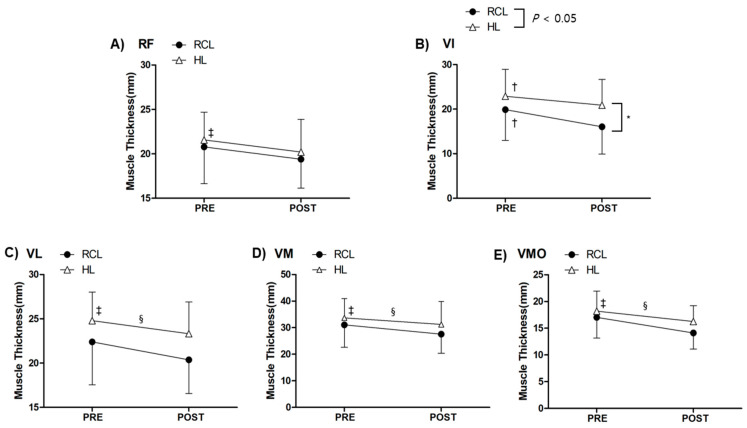
Changes in muscle thickness before and after ACLR. (**A**) Rectus femoris (RF), (**B**) Vastus intermedius (VI), (**C**) Vastus lateralis (VL), (**D**) Vastus medialis (VM), and (**E**) Vastus medialis oblique (VMO). Values are expressed as the mean ± standard error. * RCL thickness is smaller compared to HL thickness, † PRE-thickness is greater compared to POST-thickness, ‡ Average PRE-RCL and HL thickness is greater compared to average POST-RCL and HL thickness, § Average RCL thickness is smaller compared to average HL thickness.

There was a significant time–limb interaction effect for the VI (F_1,13_ = 9.325, *p* = 0.009, η^2^p = 0.418, Figure 4B). Post-reconstruction VI thickness (16.04 ± 6.13 mm) was significantly smaller compared with pre-reconstruction VI thickness (19.89 ± 6.91 mm, *p* = 0.001, Figure 4B) on RCL. In addition, post-reconstruction VI thickness (20.90 ± 5.78 mm) was significantly smaller than pre-reconstruction VI thickness (22.88 ± 6.07 mm, *p* = 0.019, Figure 4B) on HL. Post-reconstruction VI thickness on RCL (16.04 ± 6.13 mm) was significantly smaller compared with post-reconstruction VI thickness on HL (20.90 ± 5.78 mm, *p* = 0.048). We observed a time main effect on VL (F_1,13_ = 26.498, *p* = 0.001, η^2^p = 0.671, Figure 4C), VM (F_1,13_ = 27.970, *p* = 0.001, η^2^p = 0.683, Figure 4D), and VMO (F_1,13_ = 29.152, *p* = 0.001, η^2^p = 0.692, Figure 4E), suggesting that, on average, VL, VM, and VMO thickness reduced after ACLR. Additionally, there was a significant limb main effect on VL (F_1,13_ = 8.092, *p* = 0.014, η^2^p = 0.384, Figure 4C), VM (F_1,13_ = 8.254, *p* = 0.013, η^2^p = 0.388, Figure 4D), and VMO (F_1,13_ = 20.383 *p* = 0.001, η^2^p = 0.611, Figure 4E) indicating, on average, VL, VM, VMO thickness on RCL were smaller compared with HL. Lastly, there was a significant time effect only on RF thickness (F_1,13_ = 17.116, *p* = 0.001, η^2^p = 0.568, Figure 4A).

## 4. Discussion

The purpose of this study was to compare the bilateral thickness of each quadricep component before and after ACLR. While previous studies showed quadricep atrophy in ACLR patients [3,26], the instantaneous morphological alteration in each quadricep component after the surgery was unclear. The primary finding of the current investigation was a decrease in the VI thickness of the RCL within 48–72 h after the surgery, compared to the HL.

Previous investigations have reported the importance of the VI in knee function and human locomotion [13,34]. A study reported that a smaller VI thickness was associated with the knee extension torque, which is related to the performance of explosive movements [13]. In addition, another study showed that the VI is activated during the recovery phase of running, which could indicate that it provides knee joint stabilization as an antagonist during hip flexion [35]. A study with patients who had undergone ACLR showed VI thickness reduction to result in decreased knee extension torque and the same study suggested that increasing VI thickness by rehabilitation after ACLR could be the key factor for the proper restoration of knee function [36]. While the result of the current study was consistent with those of several previous studies which found reduced VI thickness after ACLR, the primary difference was the muscle thickness assessment time period after surgery. The current study measured quadricep thickness 48–72 h after ACLR, which could be considered a short period of time compared to the previous investigations (3–12 months post-ACLR). It could be a significant finding that the atrophy in specific quadricep components initiated instantaneously after ACLR. The mechanism of selective VI atrophy after ACLR is unclear; therefore, it is necessary to be further investigated for the provision of treatment options to prevent VI atrophy following surgery.

According to the current study, VI thickness in the RCL was smaller than that in the HL, after ACLR. A bilateral comparison of the quadriceps after ACLR has been considered as a standard test for decisions regarding return to play [37]. Several investigations have shown bilateral quadricep function reductions. For example, a study showed bilateral quadricep activation reduction within two years after ACLR and this activation reduction was accompanied by bilateral atrophic change in the muscle morphology [38]. Furthermore, other studies found bilateral quadricep activation deficits in patients within two years after ACL repair [2,39,40]. Compared to previous studies, the current study investigated the response of quadricep thickness to ACLR in a short period of time. We found thickness reduction in only the VI in the RCL and it could be speculated that bilateral muscle atrophy may be due to the long-term effect of the altered central nervous system on the target muscles. Therefore, it could be significant to have a proper long-term rehabilitation program to minimize bilateral quadricep atrophy after ACLR.

From a physiological point of view, changes in cytokine levels after ACLR could directly or indirectly influence the muscle size. In a previous study, myostatin, a well-known atypical atrophy-induced cytokine, increased and insulin-like growth factor 1 (IGF-1), a hypertrophy-inducing cytokine, decreased after ACLR [23]. Thus, the previous study concluded that muscle atrophy after ACLR could be influenced by these muscle morphology-related cytokines; however, they suggested further investigations since the serum biomarkers could not explain the atrophy of specific muscles such as the VI after ACLR. Although the current investigation did not analyze the atrophy-related serum cytokines, it could be speculated that the serum biomarkers were not affected by the bilateral quadricep muscle thickness in a short period of time following reconstruction. Since many researchers have reported the occurrence of bilateral muscle atrophy in patients within one year postoperatively [21,41], the alteration of bilateral quadricep morphology could be considered as the result of the long-term influence of the reconstruction. In addition, previous investigations reported the influence of tourniquet-using time to muscle atrophy because of ischemia-reperfusion injury [42,43]. These studies reported that the tourniquet-using time, which may affect to the muscle morphology, was more than 90 min [44,45]. In the current study, the average time for utilizing the tourniquet was 67 min; therefore, the influence of the tourniquet on the muscle atrophy could be minimal.

### Limitations and Future Studies

This study had several limitations. First, the patient pool in this study was not balanced (male: 10 and female: 4), as we could not match the sex of the injured patients. Second, although one surgeon completed all ACLRs, the specific surgical method and process, such as a selection of graft type and specific surgical techniques, was not controlled. Therefore, there was a possibility of surgical influence on quadricep atrophy. Third, all patients were received a general care with hospitalizing during first 48–72 h after ACLR, the activity early phase of rehabilitation was not controlled. In addition, possible pre-habilitation was not controlled. Finally, both men and women participated in the experiment, but the sex-related hormones were not controlled for.

The result of the current study indicated the muscle thickness reduction in three days after ACLR. This information could provide insights, such as that there could be unrevealed factors that influence to the muscle thickness. While many opinations regarding muscle atrophy after ACLR were focused on neuromuscular adaptation or alteration of the neuro-pathway [45,46,47,48], recent studies discussed physiological aspects of muscle atrophy after surgery [23,48]. Therefore, it could be beneficial to investigate mechanism of muscle atrophy that was influenced by surgical procedure and it could provide new therapeutic exercise and pharmacotherapeutic approach to minimize muscle atrophy during surgical procedure.

## 5. Conclusions

The current study compared the bilateral thickness of each quadricep component before and after ACLR. Only the VI in the RCL showed a significant thickness reduction compared to that in the HL. It could be speculated that the surgery influenced quadricep component thickness selectively. Further studies may be warranted to investigate the mechanism of selective quadricep atrophy and potential rehabilitation approaches to minimize selective quadriceps atrophy after ACLR.

## Figures and Tables

**Table 1:** Patient demographics.

Characteristics	
Age, years	30.4 ± 5.9
Mass, kg	69.9 ± 10.8
Height, cm	170.8 ± 8.0
Average time between injury and ACLR, days	18.3 ± 12.1
Body mass index, kg/m^2^	23.8 ± 2.3

ACLR: Anterior cruciate ligament reconstruction.
